# Direct Evidence of Endothelial Dysfunction and Glycocalyx Loss in Dermal Biopsies of Patients With Chronic Kidney Disease and Their Association With Markers of Volume Overload

**DOI:** 10.3389/fcell.2021.733015

**Published:** 2021-09-21

**Authors:** Josephine Koch, Ryanne S. Hijmans, Manuela Ossa Builes, Wendy A. Dam, Robert A. Pol, Stephan J. L. Bakker, Hendri H. Pas, Casper F. M. Franssen, Jacob van den Born

**Affiliations:** ^1^Division of Nephrology, University Medical Center Groningen, University of Groningen, Groningen, Netherlands; ^2^Department of Surgery, University Medical Center Groningen, University of Groningen, Groningen, Netherlands; ^3^Department of Dermatology, University Medical Center Groningen, University of Groningen, Groningen, Netherlands

**Keywords:** CKD, endothelial dysfunction, NT-proBNP, inflammation, sodium, glycocalyx

## Abstract

Cardiovascular morbidity is a major problem in patients with chronic kidney disease (CKD) and endothelial dysfunction (ED) is involved in its development. The luminal side of the vascular endothelium is covered by a protective endothelial glycocalyx (eGC) and indirect evidence indicates eGC loss in CKD patients. We aimed to investigate potential eGC loss and ED in skin biopsies of CKD patients and their association with inflammation and volume overload. During living kidney transplantation procedure, abdominal skin biopsies were taken from 11 patients with chronic kidney disease stage 5 of whom 4 were treated with hemodialysis and 7 did not receive dialysis treatment. Nine healthy kidney donors served as controls. Biopsies were stained and quantified for the eGC marker Ulex europaeus agglutinin-1 (UEA1) and the endothelial markers vascular endothelial growth factor-2 (VEGFR2) and von Willebrand factor (vWF) after double staining and normalization for the pan-endothelial marker cluster of differentiation 31. We also studied associations between quantified log-transformed dermal endothelial markers and plasma markers of inflammation and hydration status. Compared to healthy subjects, there was severe loss of the eGC marker UEA1 (*P* < 0.01) while VEGFR2 was increased in CKD patients, especially in those on dialysis (*P* = 0.01). For vWF, results were comparable between CKD patients and controls. Skin water content was identical in the three groups, which excluded dermal edema as an underlying cause in patients with CKD. The dermal eGC/ED markers UEA1, VEGFR2, and vWF all associated with plasma levels of NT-proBNP and sodium (all *R*^2^ > 0.29 and *P* < 0.01), except for vWF that only associated with plasma NT-proBNP. This study is the first to show direct histopathological evidence of dermal glycocalyx loss and ED in patients with CKD. In line with previous research, our results show that ED associates with markers of volume overload arguing for strict volume control in CKD patients.

## Introduction

Cardiovascular (CV) disease is the leading cause of morbidity and mortality in patients with chronic kidney disease (CKD) (Baigent et al., 2000; Matsushita et al., 2010; Mahmoodi et al., 2012; Cozzolino et al., 2017). Patients with CKD present with traditional CV risk factors, including hypertension, diabetes or dyslipidemia (Herzog et al., 2011; Gansevoort et al., 2013), but also with non-traditional CV risk factors (Go et al., 2004; El Nahas and Bello, 2005; Fox et al., 2012; Mahmoodi et al., 2012) of which endothelial dysfunction (ED) is established as a prominent one (Schächinger et al., 2000; Bonetti et al., 2003; Nagy et al., 2010).

A normally functioning endothelium modulates vascular permeability (Henry and Duling, 1999), controls vascular tone, and maintains blood flow [reviewed by Sandoo et al. (2015)]. Many of these endothelial functions are orchestrated by a properly functioning endothelial glycocalyx (eGC), which covers the luminal side of vascular endothelium. The eGC is a carbohydrate-rich mesh of glycoproteins, proteoglycans and glycosaminoglycans that interact with absorbed plasma proteins (Weinbaum et al., 2007). The highly sulfated and negatively charged properties of the eGC protect the endothelium and control cell migration (Constantinescu et al., 2003), and leakage of albumin (Ueda et al., 2004) and fluid (Van den Berg et al., 2003). Several studies indicated that damaged eGC in renal disease is associated with ED (Singh et al., 2007; Salmon et al., 2012; Vlahu et al., 2012; Padberg et al., 2014). For example, Vlahu et al. (2012) reported a diminished eGC thickness in hemodialysis patients by sidestream dark film imaging that was associated with elevated plasma markers of ED. For the development of ED, several interacting components seem to be involved. Next to oxidative stress and uremic toxins, the destructive effects of inflammation and volume overload on the vascular endothelium are increasingly receiving attention (Mitsides et al., 2018).

Patients with CKD often have elevated plasma markers of inflammation (Westphalen et al., 2020) which can have multiple underlying causes, including higher rates of various infections (De Jager et al., 2009). Additionally, accumulation of uremic toxins in patients with CKD promotes oxidative activity of leukocytes, which in turn leads to increased leukocyte-endothelial interactions, facilitating higher infiltration of macrophages and monocytes (Cozzolino et al., 2017; Liew et al., 2021). Importantly, the kidneys are a major source of antioxidants against reactive oxygen species causing diminished production of antioxidants to automatically translate in increased oxidative stress in CKD (Cozzolino et al., 2017). Next to directly inducing ED (Annuk et al., 2005), free radicals have been shown to contribute to inflammation (Kröller-Schön et al., 2014).

Overhydration, commonly seen in patients with end stage renal failure (Wizemann and Schilling, 1995; Wizemann et al., 1995), has been reported to associate with ED and microinflammation (Mitsides et al., 2017) contributing to arterial stiffness (Akdam et al., 2014), atherosclerosis, left ventricular hypertrophy (Bock and Gottlieb, 2010) and more CV events (Hung et al., 2014; Tsai et al., 2015). Interestingly, the interpretation of this association differs between authors with some concluding that ED leads to volume overload due to its role in water homeostasis (Levick and Michel, 2010; MacHnik et al., 2010; Paar et al., 2014; Dekker et al., 2017) whereas Colombo et al. posed that volume overload contributes to ED due to vascular stretch (Colombo et al., 2009, 2014). In fact, the damaging effect of overhydration on the endothelium may be due to increased shear stress as has been suggested by studies in patients with hypertension (Panza et al., 1990; Muiesan et al., 2001). Notably, endothelial cells can sense shear stress, hydrostatic pressure and stretch of the vessel wall through mechanosensors which can then be transferred into signaling pathways. Subsequently, gene and protein expression can be modified and endothelial function can deteriorate (Mammoto et al., 2012). The eGC functions as a mechanosensor of shear stress but at the same time pathologic shear stress might directly lead to eGC changes (Weinbaum et al., 2007) such as breakdown and shedding of eGC components (Zeng and Tarbell, 2014).

Clinical studies previously used indirect methods to demonstrate ED in CKD and its association with inflammation and volume overload (i.e., association analyses and imaging of the perfused boundary region) leaving open questions about possible targets for patient intervention. With the current work we aimed to investigate possible eGC loss and ED in skin biopsies of CKD patients by direct tissue staining and its association with inflammation and volume overload.

## Patients and Methods

### Study Population

During living kidney transplantation procedure, abdominal skin biopsies were taken from 11 patients with chronic kidney disease stage 5 (CKD5) of whom 4 were treated with hemodialysis (CKD5D) and 7 did not receive dialysis treatment (CKD5ND). Nine healthy kidney donors served as controls as described before (Hijmans et al., 2019). For all individuals, clinical data were obtained from electronic patient files and urine and plasma were collected on the day of the operation or 1 day before. All study participants provided written informed consent prior to study and are enrolled in the Transplantlines Biobank and Cohort study (TXLINES01). The cohort is registered at clinicaltrials.gov as NCT03272841. The study protocol is in accordance with the Dutch Medical Research Involving Human Subjects Act (WMO), and approved by the Medical Ethics Committee of the University Medical Center Groningen (METc 2014/077). All procedures were conducted in accordance with the declarations of Helsinki and Istanbul. Details on the study protocol have been described (Hijmans et al., 2019).

### Measurement of Dermal Fluid Content

The skin samples for determination of fluid content were weighed immediately after taking the biopsy, which delivered wet weight. Biopsies were then dried overnight in an oven at 80°C after which their dry weight was measured. Water content was calculated as ([wet weight – dry weight]/wet weight) × 100%. Biopsies with macroscopically visible subcutaneous fat deposits were excluded from this analysis due to disturbance of water content determination by fat deposits.

### Immunofluorescence

Immunofluorescence staining was performed on 4-μm-thick cryo sections cut from full thickness skin biopsies with a Leica CM1950 cryostat (Leica Biosystems, Wetzlar, Germany) followed by acetone fixation for 10 min. Endogenous peroxidase activity was blocked by incubating with 0.03% hydrogen peroxide (in phosphate buffered saline; PBS). Skin cryo sections were incubated for 1 h with the following primary antibodies/reagents: Rhodamine-labeled Ulex Europaeus Agglutinin I (UEA1-Rhodamine, Vector Laboratories #RL-1062), rabbit anti-human vWF (DAKO, #A0081, Santa Clara, CA, United States) and rabbit anti-human vascular endothelial growth factor receptor 2 (VEGFR2; clone D5B1, Cell Signaling Technology #9698, Danvers, MA, United States) diluted in PBS/1% Bovine Serum Albumin (BSA). Binding of primary antibodies was detected by incubating the sections for 30 min with a secondary antibody diluted in PBS/1% BSA. We used goat anti-rabbit IgG horseradish peroxidase (HRP, from DAKO) in PBS/1% BSA. Subsequently, Alexa488-conjugated mouse anti-human CD31 antibody (Abcam, ab215911, Cambridge, United Kingdom) diluted in 1% BSA was applied for 60 min. Horseradish peroxidase conjugated antibodies were visualized by the TSA TM tetramethylrhodamine system (PerkinElmer Life Sciences Inc., Waltham, MA, United States) (10 min). DAPI solution (Vector laboratories, Burlingame, CA, United States) was applied to the sections and incubated for 10 min for nuclear staining and subsequently mounted in Citifluor mounting medium (fluorescence; Sigma-Aldrich, Burlington, VT, United States). The whole staining procedure was carried out at room temperature. As negative controls, the primary antibody or lectin were replaced by PBS/1% BSA and were all found to be negative.

### Quantification of Immunofluorescence

Stainings were evaluated on a Leica DM4000B microscope (Leica Biosystems Wetzlar, Germany) equipped for immunofluorescence, and with a DFX345FX camera using a LAS software package. Five images at 20 × magnification per skin sample were taken followed by digital quantification using ImageJ 1.46r (Rasband, W.S., US National Institutes of Health). We used the pan-endothelial marker CD31 to identify vascular endothelium. To normalize for differences in vascular density and to differentiate endothelia from other cell types, the markers of interest (UEA1, VEGFR2, and vWF) were double stained with CD31. Only CD31-positive areas were quantified, and markers were expressed as a percentage/proportion of CD31-positive pixels. Subsequently, the average percentages of five images were taken to receive a single value for each patient/skin sample.

### Statistics

Data are shown as mean ± standard deviation (SD) if normally distributed and as median (interquartile range) if not normally distributed. Comparisons between groups were performed by Mann–Whitney *U* test, or Kruskal-Wallis-test if more than two groups were compared. Parameters were log-transformed for association studies using Spearman Rank correlation coefficient. Statistical analyses were performed using SPSS 25.0 (SPSS Inc., Chicago, IL, United States). *P*-values below 0.05 were considered to be statistically significant.

## Results

### Clinical Characteristics

Baseline characteristics before surgery are presented in Table 1. Among CKD patients, those with CKD5D and CKD5ND were age- and sex-matched, whereas healthy donors were ∼10 years older and comprised a higher proportion of female participants than the CKD patient group. Body mass index (BMI) and blood pressure did not differ (significantly) between the donors and patients with CKD5D or CKD5ND. The most common diseases in both CKD patient groups were IgA nephropathy, focal segmental glomerulosclerosis, autosomal-dominant polycystic kidney disease and glomerulonephritis. As expected, CKD patients had more co-morbidities compared to the donor control group, especially hypertension (36% vs. 11%), coronary heart disease (9% vs. 0%) and diabetes mellitus (9% vs. 0%). Overhydration in CKD patients was evidenced by increased values of NT-proBNP compared to controls (*P* < 0.01), both in CKD5D (*P* < 0.01) and CKD5ND patients (*P* < 0.01). In addition, fluid overload was demonstrated by decreased plasma sodium levels in CKD patients (*P* = 0.03), especially in patients with CKD5D compared to controls (*P* = 0.03). Increased CRP levels in CKD5D (*P* = 0.03) and decreased levels of plasma lymphocyte counts in CKD patients (*P* < 0.01) were seen compared to healthy subjects, especially in patients CKD5D (*P* < 0.01). This reflects an increased inflammatory status of CKD patients.

**TABLE 1:** Baseline characteristics of donors and all CKD patients (CKD5D and CKD5ND) just before surgery.

	Healthy donors (*N* = 9)	CKD patients (*N* = 11)		
		
Variables		All (*N* = 11)	CKD5D patients (*N* = 4)	CKD5ND patients (*N* = 7)
**Age (years)**	60 ± 8	51 ± 10	49 ± 10	52 ± 11
**Women, n (%)**	6 (67)	5 (46)	2 (50)	3 (43)
**BMI (kg/m^2^)**	27.1 ± 4.6	25.4 ± 3.9	28.6 ± 3.9	23.6 ± 2.7
**Blood pressure (mmHg)**				
Systolic	135 ± 16	146 ± 17	144 ± 11	147 ± 21
Diastolic	76 ± 11	82 ± 15	76 ± 18	86 ± 14
**Time on dialysis (months)**	–	–	14 (12–19)	–
**Underlying disease (%)**				
IgA nephropathy	0	18	25	14
Focal segmental glomerulosclerosis	0	9	25	0
Autosomal-dominant polycystic kidney disease	0	27	25	29
Glomerulonephritis	0	27	25	29
Other	0	19	0	28
**Known comorbidities (%)**				
Hypertension	11	36	50	29
Malignancy	11	9	0	14
Coronary heart disease	0	9	0	14
Diabetes mellitus	0	9	25	0
Other	11	19	25	14
No relevant comorbidities	67	18	0	29
**Laboratory parameters**				
Serum creatinine (μmol/L)	68 (63–83)	516 (460–655)**	811 (6045–1096)**	462 (442–516)**
eGFR (mL/min/1.73 m^2^)	91 (81–94)	11 (8–14)**	–	11 (10–14)**
Serum albumin (g/L)	44 (43–45)	43 (42–46)	43 (40–45)	43 (42–50)
Urine creatinine (mmol/L)	4 (4–16)	6 (4–9)	11 (6–11)	5 (3–8)
Proteinuria (g/L)	0.4 (0.3–0.6)	1.9 (0.3–3.4)**	2.3 (1.9–2.3)*	0.4 (0.3–6.6)
**Volume markers**				
Plasma NT-proBNP (pg/mL)	80 (28–99)	512 (282–861)**	666 (318–1481)*	399 (242–701)*
Plasma sodium (mmol/L)	141 (140–143)	139 (137–140)*	138 (137–140)*	139 (138–142)
Tissue fluid content (%)	62 (60–66)	61 (60–62)	62 (59–63)	61 (61–62)
**Inflammatory plasma markers**				
CRP (mg/l)	1.2 (0.6–1.8)	2.3 (0.5–9.0)	8.5 (4.3–9.8)*	0.6 (0.3–3.5)
Leukocytes (× 10^E^9/mL)	6.6 (6.1–7.8)	6.8 (5.5–10,0)	8.9 (6.1–10.2)	5.9 (5.4–9.6)
Monocytes (× 10^E^9/mL)	0.5 (0.5–0.6)	0.5 (0.4–0.7)	0.7 (0.4–0.9)	0.4 (0.3–0.6)
Lymphocytes (× 10^E^9/mL)	1.8 (1.8–2.2)	1.3 (1.1–1.6)**	1.5 (1.3–1.6)**	1.2 (0.9–1.4)*

*Significantly different compared to healthy donors (*P < 0.05; **P < 0.01).

### Endothelial Dysfunction in Dermal Biopsies of Patients With Renal Failure

**FIGURE 1:**
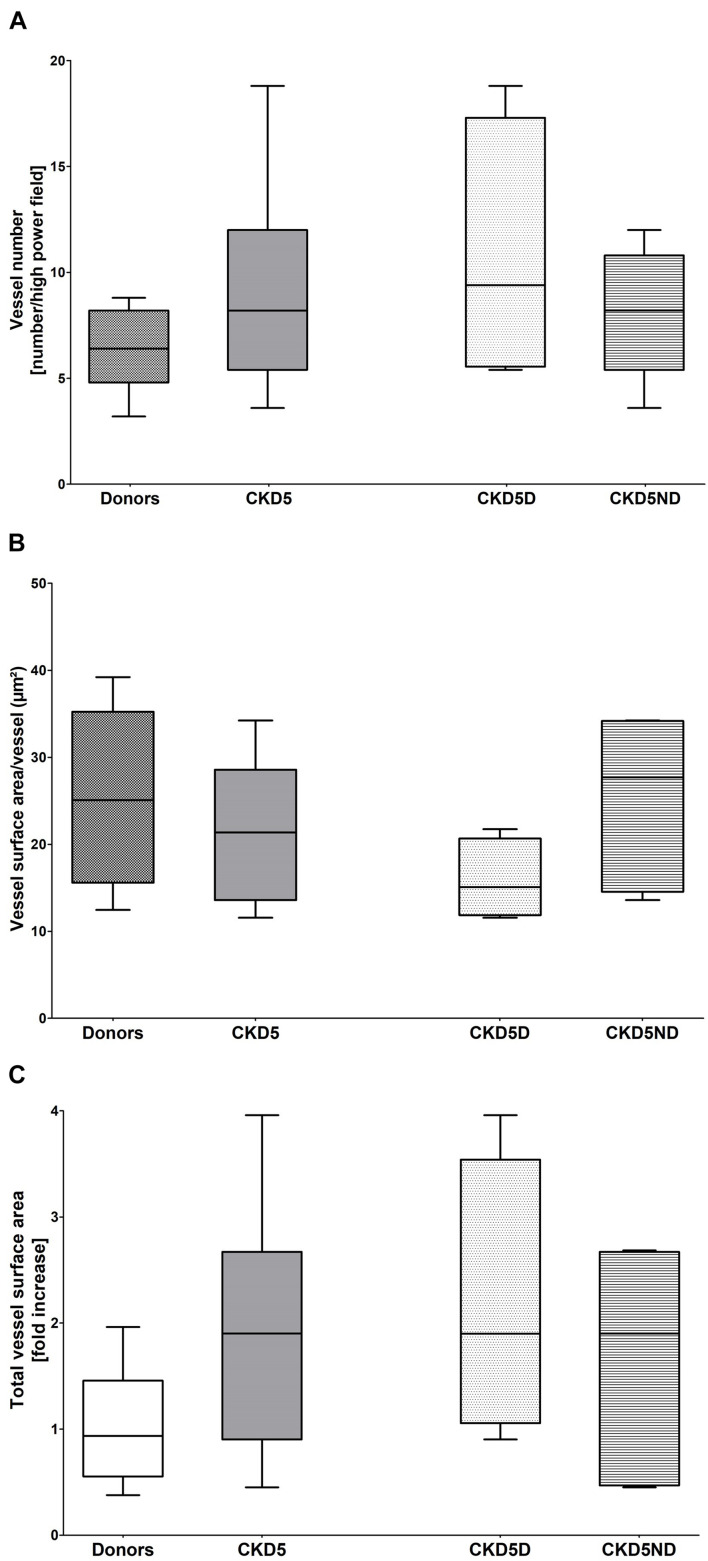
Vessel quantification based on CD31 immunofluorescence in healthy donors vs. CKD patients including CKD5ND and CKD5D patients. **(A)** Number of CD31+ vessels per group. **(B)** CD31+ vessel surface area per vessel (total CD31+ vessel surface area/vessel number) in μm^2^. **(C)** Total surface area of CD31 expression in fold increase. Differences are not statistically significant.

The analysis of vessel number, vessel area/vessel number and total vessel area (CD31) revealed no significant differences between the three groups (Figures 1A–C), indicating neither dermal angiogenesis nor hyperaemia in CKD patients. Values of dermal water content were comparable between groups, showing no dermal edema in CKD patients (Table 1).

**FIGURE 2:**
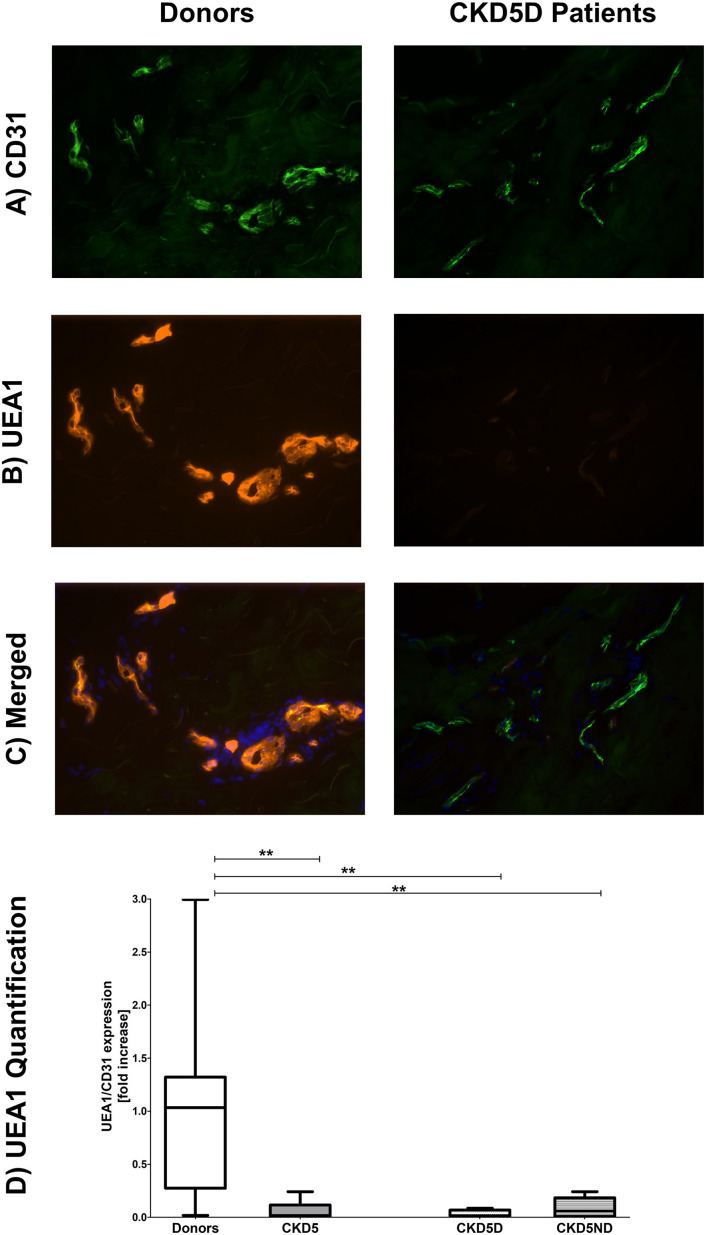
Expression of UEA1 in the microvasculature of dermal biopsies patients with CKD. **(A)** Expression of CD31 in donors (left image) and CKD5D patients (right image). **(B)** Expression of UEA1 in donors (left image) and CKD5D patients (right image). **(C)** Merged expression of UEA1 and CD31 in donors (left image) and CKD5D patients (right image). **(D)** UEA1 quantification relative to CD31. Colors in images: green = CD31; red = UEA1; blue = DAPI. Statistical significance: **P < 0.01.

To correct for apparent differences in vascular surface area between samples and to distinguish endothelia from other cell types, we exclusively evaluated the endothelial markers in a double staining with CD31. The endothelium in skin biopsies of CKD patients showed severe loss of the eGC marker UEA1 as compared to healthy controls (*P* < 0.01). Upon separation of the CKD patients, CKD5D patients (*P* < 0.01) as well as CKD5ND patients (*P* < 0.01) showed a severe decline in UEA1 compared to controls. Representative photomicrographs and results of quantitative analyses are shown in Figure 2.

**FIGURE 3:**
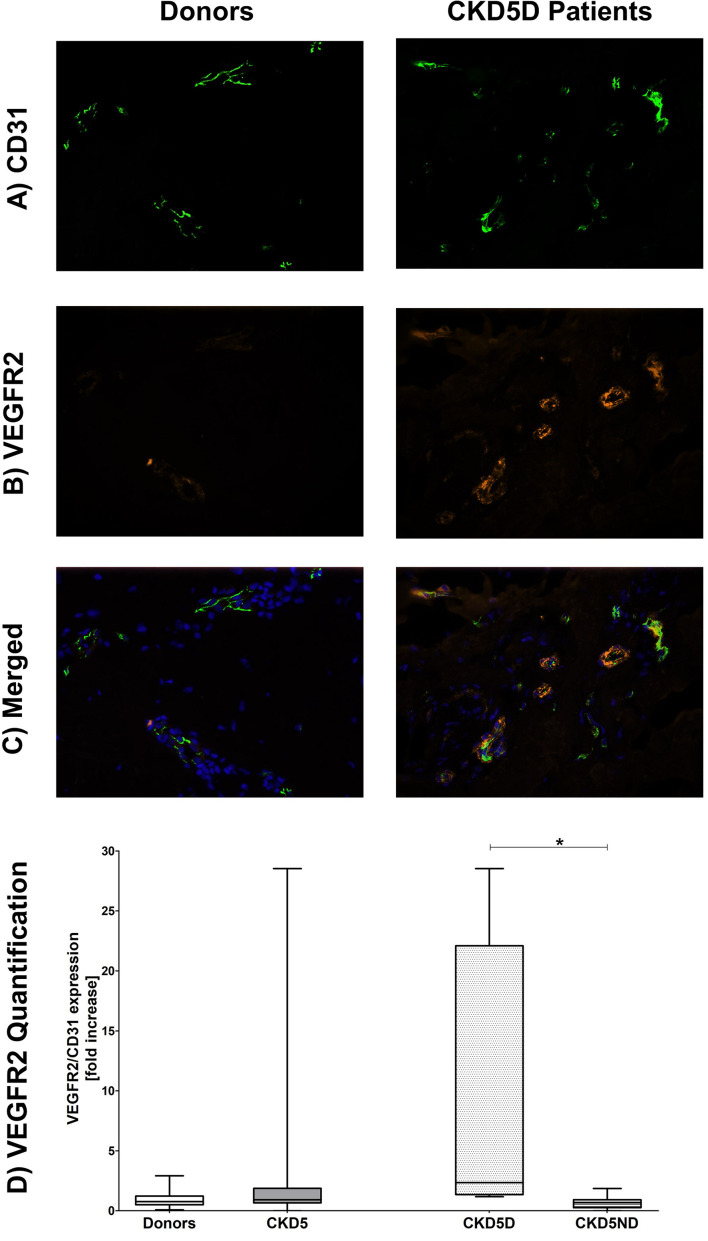
Expression of VEGFR2 in the microvasculature of dermal biopsies in patients with CKD. **(A)** Expression of CD31 in donors (left image) and CKD5D patients (right image). **(B)** Expression of VEGFR2 in donors (left image) and CKD5D patients (right image). **(C)** Merged expression of VEGFR2 and CD31 in donors (left image) and CKD5D patients (right image). **(D)** VEGFR2 quantification with VEGFR2 expression relative to CD31. Colors in images: green = CD31; red = VEGFR2; blue = DAPI. Statistical significance: *P < 0.05.

Quantification of endothelial VEGFR2 expression showed a small increase in CKD patients compared to healthy individuals, although not statistically significant for the whole CKD group (*P* = 0.65). When looking closer, only CKD5D patients appeared to have this rise with a borderline significance compared to controls (*P* = 0.05). CKD5D patients had significantly higher VEGFR2 expression than CKD5ND patients, indicating endothelial activation (*P* < 0.05) (Figure 3).

**FIGURE 4:**
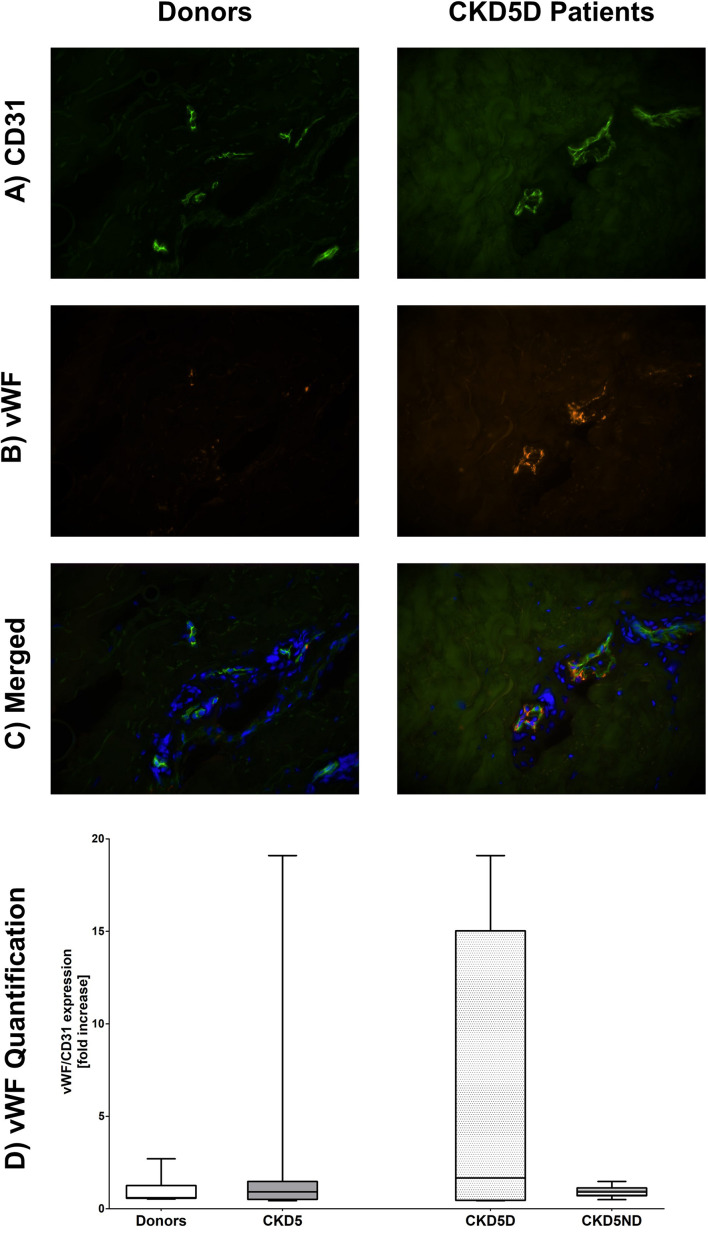
Expression of vWF in the microvasculature of dermal biopsies in patients with CKD. **(A)** Expression of CD31 in donors (left image) and CKD5D patients (right image). **(B)** Expression of vWF in donors (left image) and CKD5D patients (right image). **(C)** Merged expression of vWF and CD31 in donors (left image) and CKD5D patients (right image). **(D)** vWF quantification with vWF expression relative to CD31. Colors in images: green = CD31; red = vWF; blue = DAPI. Differences between groups are not statistically significant.

For the endothelial marker vWF, only one patient showed very high levels whereas all other CKD patients had rather similar values in comparison to controls (*P* = 0.82; Figure 4).

### Endothelial Dysfunction Associates With Volume Overload Rather Than Inflammation

**FIGURE 5:**
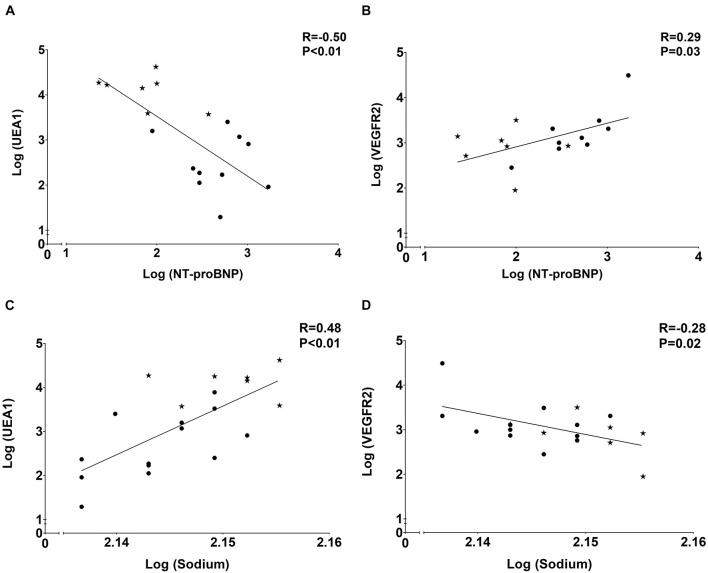
Associations of plasma volume markerswith tissue endothelial markers. **(A)** Negative association of eGC marker UEA1 and volume marker NT-proBNP. **(B)** Positive association of endothelial marker VEGFR2 and volume marker NT-proBNP. **(C)** Positive association of eGC marker UEA1 and volume marker sodium. **(D)** Negative association of endothelial marker VEGFR2 and volume marker sodium. Dots represent CKD5 patients and stars indicate controls.

As presented in Table 2 and Figure 5, the tissue markers of ED UEA1, VEGFR2, and vWF were associated with plasma markers of overhydration. The volume marker NT-proBNP inversely associated with UEA1 (*R* = −0.50; *P* < 0.01; Figure 5A). NT-proBNP also correlated significantly with VEGFR2 (*R* = 0.29; *P* = 0.03; Figure 5B) and with vWF (*R* = 0.29; *P* = 0.03). Plasma levels of sodium (with lower levels suggesting volume overload) significantly associated with the endothelial tissue marker UEA1 (*R* = 0.69; *P* < 0.01; Figure 5C) and showed an inverse relationship with tissue VEGFR2 (*R* = −0.54; *P* = 0.01; Figure 5D).

**TABLE 2:** Linear regression analysis of quantified tissue endothelial markers and laboratory parameters with plasma markers for volume and inflammation.

	**Plasma markers of volume overload**		**Plasma markers of inflammation**	
		
	**NT-proBNP (↑)**	**Sodium (↓)**	**CRP (↑)**	**Lymphocytes (↓)**
**Tissue endothelial markers**				
UEA1 (↓)	***R = −0.50; P < 0.01***	***R = 0.69; P < 0.001***	***R = −0.44; P = 0.07***	***R = 0.69; P = 0.002***
VEGFR2 (↑)	***R = 0.29; P = 0.03***	***R = −0.54; P < = 0.05***	*R* = 0.11; *P* = 0.24	***R = 0.34; P = 0.61***
vWF (↑)	***R = 0.29; P = 0.03***	*R* = 0.28; *P* = 0.54	*R* = 0.11; *P* = 0.90	*R* = 0.26; *P* = 0.18
**Patient characteristics**				
Age	*R* = *−*0.38; *P* = 0.16	*R* = 0.11; *P* = 0.66	*R* = 0.15; *P* = 0.56	*R* = 0.04; *P* = 0.89
BMI	*R* = 0.05; *P* = 0.86	*R* = *−*0.36; *P* = 0.14	*R* = *−*0.29; *P* = 0.26	*R* = *−*0.39; *P* = 0.14
Systolic blood pressure	*R* = 0.18; *P* = 0.53	*R* = *−*0.17; *P* = 0.50	*R* = *−*0.25; *P* = 0.33	*R* = *−*0.47; *P* = 0.06
Diastolic blood pressure	*R* = 0.20; *P* = 0.48	*R* = 0.07; *P* = 0.79	*R* = 0.10; *P* = 0.72	*R* = 0.07; *P* = 0.80
**Laboratory parameters**				
Serum creatinine	***R = 0.68 P < 0.01***	*R* = *−*0.27 *P* = 0.27	*R* = *−*0.25 *P* = 0.34	***R = −0.71 P < 0.01***
eGFR	***R = −0.63 P = 0.02***	*R* = 0.09 *P* = 0.73	*R* = *−*0.04 *P* = 0.99	***R = 0.58 P = 0.03***
Serum albumin	*R* = *−*0.15 *P* = 0.60	*R* = 0.32 *P* = 0.20	*R* = *−*0.16 *P* = 0.56	*R* = *−*0.27 *P* = 0.33
Urine creatinine	***R = −0.68 P < 0.01***	*R* = −0.71 *P* = 0.27	***R = −0.25 P < 0.01***	***R = −0.71 P < 0.01***
Proteinuria	*R* = 0.42 *P* = 0.16	***R = −0.71 P < 0.01***	*R* = *−*0.20 *P* = 0.50	***R = −0.70 P = 0.01***
**Markers of volume overload**				
Plasma NT-proBNP	–	***R = −0.45; P = 0.04***	***R = 0.56***;***P = 0.01***	***R = −0.60; P < 0.01***
Plasma sodium	–	–	*R* = *−*0.33; *P* = 0.14	***R = 0.64; P < 0.01***
Tissue fluid content	*R* = 0.35 *P* = 0.17	*R* = *−*0.06 *P* = 0.81	*R* = 0.43 *P* = 0.07	*R* = 0.07 *P* = 0.79
**Plasma markers of inflammation**				
CRP	***R = 0.56 P = 0.01***	*R* = *−*0.33 *P* = 0.14	–	*R* = *−*0.19 *P* = 0.42
Leukocytes	*R* = 0.13 *P* = 0.59	*R* = 0.26 *P* = 0.22	*R* = 0.26 *P* = 0.25	*R* = 0.36 *P* = 0.12
Monocytes	*R* = 0.05 *P* = 0.83	*R* = 0.07 *P* = 0.78	*R* = *−*0.13 *P* = 0.60	*R* = 0.26 *P* = 0.27
Lymphocytes	***R = −0.61 P < 0.01***	***R = 0.64 P < 0.01***	*R* = *−*0.19 *P* = 0.42	–

*Arrows next to parameters indicate the direction of value change in CKD patients. Significant results are shown in bold italics.*

Plasma markers of inflammation did barely associate with endothelial tissue markers (Table 2), except for plasma lymphocytes correlating with UEA1 (*R* = 0.69; *P* < 0.01). Additionally, we found significant associations between NT-proBNP and CRP (*R* = 0.31; *P* = 0.01), NT-proBNP and blood lymphocytes (*R* = −0.60; *P* < 0.01) and between plasma sodium and blood lymphocyte number (*R* = 0.64; *P* < 0.01) showing a link between volume and inflammation parameters.

## Discussion

This study is the first to show direct evidence of dermal glycocalyx loss and ED in patients with CKD. In line with previous research, our results show that ED associates with volume overload rather than inflammation, emphasizing the importance of strict volume control in patients with CKD5. In fact, ED was associated with severe loss of the eGC marker UEA1 in endothelial cells of the dermal microcirculation of patients with CKD stage 5. Moreover, we found increased dermal endothelial expression levels of VEGFR2 in hemodialysis patients. A clear association between ED and volume overload was evidenced by elevated plasma levels of NT-proBNP and reduced plasma levels of sodium in CKD5 patients that significantly correlated with the tissue endothelial markers UEA1 and VEGFR2; and tissue vWF associating with plasma levels of NT-proBNP. We hardly found associations between plasma markers of inflammation and markers of ED in dermal biopsies. Together, these results suggest that dermal ED in CKD patients is associated with overhydration rather than with inflammation.

Chronic kidney disease stage 5 patients in our cohort clearly showed ED as evidenced by decreased UEA1 expression, and increased tissue VEGFR2 and vWF compared to healthy subjects. In this study, especially the breakdown of the eGC in CKD5 patients was striking as indicated by severe loss of the UEA1 glyco-epitope. Breakdown of eGC was significantly present in both CKD patient groups, but showed a tendency to be higher in patients with CKD5D than in those with CKD5ND. Breakdown of the eGC in CKD patients has been shown before by several indirect methods. Vlahu et al. (2012) used sidestream darkfield imaging to visualize the sublingual microcirculation. Other study groups showed similar results using the same technique in CKD patients (Dane et al., 2014; Mitsides et al., 2018). Several more publications reported glycocalyx components such as hyaluronan and syndecan-1 in plasma to be associated with eGC breakdown in CKD patients (Padberg et al., 2014; Liew et al., 2021). Moreover, Padberg et al. (2014) used atomic force microscopy to directly measure eGC in a CKD rat model. Here, they found decreased eGC thickness and stiffness which correlated with plasma biomarkers of ED such as vWF (Padberg et al., 2014).

In this study, we found strong associations between ED and volume overload in CKD patients. Increased plasma levels of NT-proBNP were present in all CKD patients as well as reduced sodium values, most outspoken in patients with CKD5D. NT-proBNP significantly associated with the tissue endothelial markers UEA1, VEGFR2, and vWF whereas plasma sodium associated with UEA1 and VEGFR2. The association between ED and volume overload has been shown before by several other groups that reported breakdown of the endothelial glycocalyx (Chappell et al., 2014; Mitsides et al., 2017) and elevated plasma levels of the endothelial markers vascular cell adhesion protein 1 (VCAM-1) and thrombomodulin (Mitsides et al., 2017). Indeed, Chappell et al. observed that inducing acute hypervolemia by infusing 20 mL/kg body weight of an iso-oncotic hydroxyethyl starch (HES) colloid solution (6% HES 130/0.4, Volulyte) led to shedding of the endothelial glycocalyx as evidenced by a significant increase in plasma levels of atrial natriuretic peptide and a rise in serum hyaluronan and serum and urine syndecan-1 (Chappell et al., 2014). In our study, we did not find differences between CKD patients and healthy controls with regard to dermal fluid content. Thus, we can only assume that volume overload was predominantly intravascular. We speculate that this could have led to vascular stretch and, subsequently, to ED. Previous research showed endothelial cell activation after vascular stretch from acute volume loading (central venous pressure ≥20 mmHg). After that, the vascular endothelium of healthy dogs showed increased mRNA levels of cyclooxygenase 2 and induced nitric oxide synthase which resulted from activation of the inflammatory/oxidative and hemostatic programs, similar to dogs suffering from heart failure (Colombo et al., 2009). More studies indicated ED and subsequent atherosclerosis after excessive vascular stretch (Hatami et al., 2013) as it is seen in hypertension, heart and kidney failure. Here, vascular remodeling took place after pathologic stretch with subsequent cell apoptosis, inflammation and the production of reactive oxygen species (Okada et al., 1998; Lemarié et al., 2010) and [reviewed by Jufri et al. (2015)]. In addition, cell differentiation from endothelial cell to smooth muscle cell has been observed after application of vascular stretch (Cevallos et al., 2006). In our cohort, endothelial VEGFR2 expression was significantly upregulated in dialysis patients. It has been demonstrated that VEGFR2 can get activated upon binding with its ligands VEGFA, VEGFC or VEGFD leading to endothelial cell proliferation, angiogenesis, cell survival but also to vascular permeability [reviewed by Lohela et al. (2009)]. Our study could neither show signs of angiogenesis nor of dermal edema. Nevertheless, higher values of VEGFR2 were only seen in CKD5D patients and a direct association between NT-proBNP and VEGFR2 was observed. It has been shown that stretching of the vasculature might directly induce VEGFR2 expression which can be interpreted as an endothelial stress response (Smith et al., 2001; Tian et al., 2016).

According to our data, dialysis patients showing eGC damage had significantly increased CRP values. In a previous publication on this cohort, we showed increased inflammation in dermal biopsies of CKD5D patients with increased levels of tissue macrophages and T-cells. Besides, dermal endothelial stainings of the chemokine C-C Motif Chemokine Ligand 2 (CCL2) and hyaluronan were performed. Moreover, we performed quantitative RT-PCR on CCL2, VCAM-1, and three hyaluronan enzymes. These data showed significant loss of hyaluronan staining, which is in full agreement with our data on UEA1 in the current report. Moreover, we showed a significant inverse association of quantitative mRNA expression of hyaluronan synthase 2 with the quantitative mRNA expression of CCL2. These data suggest that dermal inflammation in CKD5 patients is quantitatively associated with loss of hyaluronan (Hijmans et al., 2019). In the present study we found an association between plasma lymphocytes and tissue UEA1 suggesting an association between glycocalyx breakdown and lymphocytopenia, thus with inflammation which is in accordance with published research (Moseley et al., 1997; Vigetti et al., 2010; Oberleithner et al., 2011; Vlahu et al., 2012). In the (above-mentioned) study of Vlahu et al. (2012), the eGC breakdown was indeed related to inflammation in dialysis patients with elevated CRP values (>10 mg/L) having significantly increased perfused boundary regions. Besides, Kusche-Vihrog et al. (2011) have directly measured eGC stiffness of human endothelial cells (Eahy.924) by atomic force microscopy and demonstrated an increase in eGC stiffness after incubation with CRP. Subsequently, fluid permeability increased (Kusche-Vihrog et al., 2011). More *in vitro* studies reported reactive oxygen species and inflammatory cytokines such as tumor necrosis factor-α to lead to hyaluronan degradation in the glycocalyx of endothelial cells and polymorphonuclear leukocytes (Moseley et al., 1997; Vigetti et al., 2010). Importantly, inflammation might also be linked to (and probably induced by) overhydration as has been shown by this work and other researchers [reviewed by Colombo et al. (2008)]. Our study showed signs of generalized inflammation with increased CRP and lymphocytopenia, with the latter being associated with eGC breakdown. Thus, although the associations of increased vascular volume with ED are much stronger, we cannot exclude the potential contribution of a chronic inflammatory state to ED in patients with CKD.

This study has several limitations. Firstly, as we observed more co-morbidities such as hypertension, coronary heart disease or diabetes in CKD patients compared to healthy controls, we cannot exclude that our results were influenced by a difference in comorbidity burden. Nevertheless, high blood pressure seemed to be treated quite well considering the comparable blood pressure between CKD patients and healthy donors. Moreover, there was only one diabetic CKD patient in the CKD study group. Secondly, we observed variability within each group which can reflect biological variability in microvascular density and/or sampling error. Lastly, we only had access to a small number of patients which limits the validity of our results.

To conclude, this study is the first to show direct evidence of dermal glycocalyx loss and ED in patients with CKD. Moreover, plasma markers of volume overload were associated with tissue markers of endothelial breakdown (i.e., glycocalyx) and endothelial activation (i.e., VEGFR2 and vWF). Although our results argue for strict volume control in CKD patients, future studies should address whether such an approach is capable of attenuating the eGC loss and ED in these patients.

## Data Availability Statement

The original contributions presented in the study are included in the article/supplementary material, further inquiries can be directed to the corresponding author.

## Ethics Statement

The studies involving human participants were reviewed and approved by the study protocol is in accordance with the Dutch Medical Research Involving Human Subjects Act (WMO), and approved by the Medical Ethics Committee of the University Medical Center Groningen (METc 2014/077). All procedures were conducted in accordance with the declarations of Helsinki and Istanbul. Details on the study protocol have been described (Hijmans et al., 2019). The patients/participants provided their written informed consent to participate in this study.

## Author Contributions

JB, CF, RP, SB, and HP conceived and designed the study and made the skin biopsies and clinical data available. JK, RH, MO, and WD performed the laboratory measurements. JK, JB, and CF analyzed and interpreted the data and drafted the manuscript. All authors approved the final version of the manuscript.

## Conflict of Interest

The authors declare that the research was conducted in the absence of any commercial or financial relationships that could be construed as a potential conflict of interest.

## Publisher’s Note

All claims expressed in this article are solely those of the authors and do not necessarily represent those of their affiliated organizations, or those of the publisher, the editors and the reviewers. Any product that may be evaluated in this article, or claim that may be made by its manufacturer, is not guaranteed or endorsed by the publisher.

## Abbreviations

BSA: bovine serum albumin
ED: endothelial dysfunction
eGC: endothelial glycocalyx
CD31: cluster of differentiation 31
CCL2: C-C Motif Chemokine Ligand 2
CKD: chronic kidney disease
CKD5: chronic kidney disease stage 5
CKD5D: chronic kidney disease stage 5 on dialysis
CKD5ND: chronic kidney disease stage 5 not on dialysis
CV: cardiovascular
HES: hydroxyethyl starch
NT-proBNP: N-terminal prohormone of brain natriuretic peptide
PBS: phosphate-buffered saline
UEA1: Ulex europaeus agglutinin-1
VCAM-1: vascular cell adhesion protein 1
VEGFR2: vascular endothelial growth factor receptor-2
vWF: von Willebrand factor.
